# From promise to practice: the growth and future of individual placement and support employment support in Aotearoa New Zealand

**DOI:** 10.3389/phrs.2026.1609475

**Published:** 2026-10-22

**Authors:** Helen Lockett, Warren Elwin, Robert Williams

**Affiliations:** 1 Wise Group, Hamilton, New Zealand; 2 University of Otago Wellington, Wellington, New Zealand; 3 Work Counts, Hamilton, New Zealand

**Keywords:** Aotearoa New Zealand, equity, indigenous leadership, individual placement and support, policy, evidence-based practices, scale-up

## Abstract

**Background:**

This policy brief examines Individual Placement and Support (IPS) in Aotearoa New Zealand since 2019, within wider health, welfare, and labour market settings.

**Analysis:**

Using a macro (policy, funding), meso (organisational and services) and micro (practitioner level) framework, IPS availability and implementation quality are examined. The IPS workforce has doubled in 5 years to 113 full-time equivalent employment specialists across 13 of 19 health districts. However, service quality, as measured by fidelity score is variable, and access is uneven.

**Policy options:**

IPS already exists, the workforce and implementation infrastructure exists, and outcomes have been demonstrated. New Zealand has solved the effectiveness problem, but not the authorisation one. Clear accountability for investing in evidence-based employment services for people in contact with specialist mental health and addiction services is needed from government agencies. Drawing on research, fidelity reviews, and national policy, recommendations are made to support equitable scale-up.

**Conclusion:**

IPS employment support is simultaneously an employment and a mental health intervention, as well as a mechanism to address systemic inequities. There is ample, robust evidence that IPS works in New Zealand. What is needed is a system that is accountable for delivering it at scale.

## Background

Employment is a key determinant of health and wellbeing, and unemployment is frequently a consequence following the onset of a mental health condition [1]. For people experiencing mental health conditions being supported in specialist mental health and addiction services, the proportion employed is substantially lower than the general population [2], despite many expressing the desire to work and resume careers.

Individual placement and support (IPS) is the internationally recognised, evidence-based practice to support people with mental health conditions to gain and sustain employment. Where IPS is offered, people’s chances of returning to employment significantly increase [3].

Individual Placement and Support services have been actively implemented in Aotearoa New Zealand (NZ) since the early 2000s, initially through pilot programs integrating employment specialists into adult community mental health teams. These pilots demonstrated improved outcomes when employment support is embedded within clinical care. Subsequent evaluations assessed early implementation, fidelity, and outcomes and identified factors helping and hindering IPS implementation in the Aotearoa NZ context [4–6].

Although IPS has been implemented successfully in several health districts in New Zealand, access remains limited and very uneven. Scaling up IPS nationally presents an opportunity to honour lived experience aspirations, support recovery-oriented mental health services, and contribute to broader societal issues related to equity, population wellbeing and labour force participation. IPS represents one of the few mental health interventions for which effectiveness, implementation feasibility, and economic benefits have all been demonstrated.

This policy brief builds on previous New Zealand IPS policy analysis, and considers the current policy landscape, coverage and quality of IPS services to make recommendations to inform a successful policy roadmap to achieve national scale-up.

### The Aotearoa New Zealand context

Aotearoa New Zealand, a South Pacific nation of around 5.3 million people, spans two main islands, Te Ika-a-Māui (North Island) and Te Waipounamu (South Island), with diverse populations and needs across urban and rural areas. Approximately 18% of the population identify as Māori (tāngata whenua, Indigenous people), 17% as Asian, and 9% as Pacific peoples. Nearly 16% of the population live in rural areas [7, 8].

Te Tiriti o Waitangi (the Treaty of Waitangi, 1840) affirms Māori sovereignty, land rights, and cultural identity. It remains foundational to New Zealand’s governance and guides efforts toward equitable, Treaty-aligned service design.

Recent health reforms have centralised service delivery under Te Whatu Ora (Health New Zealand), replacing 20 District Health Boards. Te Whatu Ora (Health NZ) is responsible for purchasing and providing mental health and addiction services (either directly or contracting non-government providers). Health NZ is divided into four Health regions and 19 Health Districts [9]. Health policy leadership remains with Manatū Hauora (Ministry of Health).

Te Aka Whai Ora (the Māori Health Authority) was established in 2022 to strengthen Māori health outcomes and Te Tiriti o Waitangi partnerships. Te Aka Whai Ora was disestablished in 2024, with functions transferred to Te Whatu Ora (Health NZ) and Manatū Hauora (Ministry of Health) [10]. The creation of New Zealand’s first Minister for Mental Health in 2023 signalled renewed political commitment to mental health and addiction service access and workforce growth.

Labour market indicators indicate inequities. As of June 2025, Māori employment rates were 62.2%, compared with 74.3% for New Zealand Europeans [11, 12]. The Ministry of Social Development (MSD) is responsible for providing active labour market programs and administering core welfare benefits through Work and Income New Zealand [13]. These welfare benefits include Jobseeker Support, Sole Parent Benefit, and Supported Living Payment. Supported Living Payment is available for people who are disabled, or who have a health condition or injury that has a severe and permanent impact on a person’s ability to work. At September 2025, of the 106,890 Supported Living Payment recipients, 36.5% are registered as having mental health conditions as the primary reason for claiming benefits [14]. Māori are disproportionately likely to be recipients of supported living payment for mental health reasons [12, 14].

## Analysis

### IPS coverage in Aotearoa NZ

By 2019, approximately 52.7 full-time equivalent (FTE) employment specialists were operating nationwide, a reach of 3.7 FTE per 10,000 adults accessing specialist mental health and addiction services [15]. In New Zealand, approximately 3 percent of the adult population access specialist services, so this equates to 3.7 FTE per 333,333 adults in the general population [16].

By 2026, Work Counts, the national centre for IPS implementation, reported 113.4 full-time equivalent (FTE) employment specialists across 82 clinical teams in 13 health districts, more than double the 2019 levels [15, 17]. Assuming average caseloads of 20 people per employment specialists turning over once per year (i.e. 40 people supported per employment specialist per year). This suggests IPS support for approximately 4,500 individuals annually, only 3% of the adults accessing specialist mental health and addiction services [16].

The growth in IPS programs has been concentrated in Auckland (from 17 to 42.5 FTEs) and Northland (from 2 to 10 FTEs), with smaller expansions in the Nelson Marlborough health district, and new expansion into the West Coast district. Some IPS practice has also extended into Housing First and addiction services. However, districts without IPS in 2019 largely remain without access, and rural inequities persist (see Table 1).

**TABLE 1:** A comparison of full-time equivalent (FTE) employment specialists by Aotearoa NZ health district in 2019 and 2026.

Health district	IPS FTE 2019^2^	IPS FTE^1^ 2026^3^	Health funded FTE 2026	MSD^3^ funded FTE 2026	Non-government funded FTE 2026
Northland - Te Tai Tokerau	2	10	✓	✓	x
Auckland	5	13	✓	✓	x
North Shore, Waitākere, Rodney	8	16.5	✓	✓	x
Counties Manukau	4	13	✓	✓	✓
Waikato	10.5	12	✓	x	✓
Taranaki	5.2	6	✓	✓	x
Rotorua and Taupō	5	5	✓	x	x
Bay of Plenty	0	1	x	x	✓
Nelson Marlborough	2	6.8	✓	x	x
Canterbury	1	13	x	✓	x
Wellington, Kāpiti and Hutt Valley	9	12.1	✓	x	x
West Coast	0	3	✓	x	x
Whanganui	1	2	✓	✓	x
**Total**	**52.7**	**113.4**	​	​	​

1. FTEs are included where the contract for support services stimulates IPS, or the provider has undertaken IPS training, or had a fidelity review in the last 2 years 2. Source: Cram F, Jury S, Kokaua J, et al. Individual Placement and Support (IPS) in Aotearoa New Zealand–new insights from linked administrative data [Internet]. Wellington (NZ): Ministry of Social Development; 2020 [cited 2025 Dec]. Available from: https://www.msd.govt.nz/documents/about-msd-and-our-work/publications-resources/research/ individual-placement-and-support/ips-new-findings-report.pdf. 3. Source: Work Counts. IPS employment support in Aotearoa New Zealand [Internet]. Hamilton (NZ): Work Counts; 2026 [cited 2026 Sep]. Available from: https://www.workcounts.co.nz/library/ips-coverage-map‐2026/. 4. Bold figures are the total IPS full time equivalents employment specialists in Aotearoa NZ in 2019 and 2026.

New providers have entered the ecosystem, including Māori-led mental health and addiction providers, and Odyssey, specialising in addiction recovery, illustrating increased diversity in IPS leadership, capability and reach [17].

So, whilst growth in the IPS workforce in New Zealand in the last 5 years has been substantial, it remains uneven and absent in many health districts, highlighting challenges of equitable access.

### The authorising system for IPS implementation

Both local and international evidence identifies enablers of IPS quality and availability across countries. These factors include the call from people with lived experience to respond to their employment aspirations, aligned funding and policy across government departments, the availability and funding of technical assistance and training, indigenous leadership, and an explicit and intentional focus on equity. At the same time, growth depends on clinical and employment service collaboration, local champions, local research evidence, and participation in regional and international IPS learning communities [4–6, 18–20].

A multi-level framework, macro (policy and funding), meso (organisational systems), and micro (frontline), helps illuminate and categorise the factors influencing the quality of collaboration needed for IPS implementation and scale-up - the IPS authorising system.

Examination of this system can help to identify policy options and recommendations for national scale‐up and equitable coverage. Macro factors include funding, financing and resourcing, government policies and legislation and regulatory frameworks. Meso factors include those factors which support health, welfare and employment organisations to collaborate and to integrate services, components essential for the delivery of high fidelity IPS programs. These factors include but are not limited to shared vision and objectives across agencies and organisations, management and leadership, training and technical assistance, shared information and referral systems. Micro factors are those at a practitioner level including values alignment and beliefs, sharing information and role and responsibilities [19].

### Macro factors

In 2018, two significant and complementary reviews of the mental health and addiction system were published. The Government Inquiry into mental health and addiction services: He Ara Oranga, and the OECD Mental Health and Work NZ review [1, 21]. Both acknowledged the central role of employment to mental health and wellbeing and the importance of increasing the availability of, and access to integrated employment and mental health services, particularly IPS programs.

Subsequent cross-government mental health, welfare and disability strategies have also acknowledged employment as central to mental health and wellbeing and identified IPS as a core service in a contemporary mental health and addiction system [22–26].

In August 2026, the Minister for Mental Health launched the 10-year strategy for mental health and wellbeing [25]. In the Strategy and accompanying three-year Implementation plan, there is a commitment to doing more of what works. This includes exploring opportunities to stabilise and scale evidence-based integrated services across mental health and addiction, employment and housing, and ensuring people engaged with, or transitioning from, specialist mental health and addiction services receive integrated support for employment [25].

Whilst all these policies signal a supportive direction, neither Health NZ performance frameworks, or MSD active labour market programs currently hold agencies accountable for competitive employment outcomes among people engaged with specialist mental health and addiction services. Whilst some joint funding from Te Whatu Ora (Health NZ) and the MSD has enabled regional innovation, particularly in Auckland and Northland, national scale-up requires pooled commissioning and stable, multi-year investment [2, 6, 18, 20].

Between 2018 and 2023, a national IPS steering group developed *Honouring Aspirations,* a plan to scale up IPS nationally, and supported the first co-funded programs through Vote Health and Vote Social Development [27]. Although the national group disbanded in 2023, regional collaborations, such as in Northland, appear to have sustained progress. Māori participants account for nearly half of successful job placements in that health district, likely related to Māori leadership across clinical and employment services, and an explicit focus on equity [6, 28].

### Meso factors

Implementation support, with access to regular fidelity reviews, is a major driver of quality and consistency in IPS programs [29]. Work Counts, established in 2018, provides national coordination, technical assistance, fidelity reviews, workforce training, and access to the International IPS Learning Community. These supports foster shared learning and enable consistent application of core IPS principles.

Between June 2020 and June 2026 Work Counts conducted 38 fidelity reviews (Table 2). Scores ranged from 52/125 (not IPS) to 114/125 (good fidelity), with 14 reviews scoring good fidelity. Most sites scored 4 or 5 on the staffing items, integration with mental health teams, the role of the supervisor, rapid job search and diversity of jobs, employers and a focus on competitive employment. The lowest-scoring items, representing areas for improvements for clinical teams are: zero exclusion, the focus on competitive employment, and executive team support. This includes including routinely asking people about employment aspirations when they first access mental health and addiction services and the measurement of employment aspirations and outcomes in health records. For the employment teams, areas for improvement include: frequency of contact with employers for job development, the provision of ongoing support once someone secures a job and time spent in the community.

**TABLE 2:** Fidelity scores - reviews conducted between June 2020 and June 2026, using the 25-item fidelity scale^1^ (n = 38).

Staffing Items^2^			Sub-total	Organisation Items^3^					​	​	​	Sub-total
1	2	3	​	4	5	6	7	8	9	10	11	​
5	5	5	15	5	4	5	5	3	3	3	3	31
4	5	5	14	5	4	3	5	4	3	2	2	28
5	5	5	15	5	5	4	4	2	5	4	4	33
4	5	5	14	5	5	4	4	2	3	4	5	32
5	5	5	15	5	4	3	5	3	3	3	4	30
5	5	5	15	3	4	4	3	3	3	1	4	25
4	5	5	14	3	4	3	3	5	3	3	4	28
5	5	4	14	4	4	2	5	4	5	4	4	32
5	5	5	15	5	5	4	4	2	2	2	4	28
5	5	5	15	4	3	3	3	4	5	3	4	29
5	5	5	15	5	5	2	5	5	5	1	4	32
5	5	5	15	5	3	3	5	4	5	2	4	31
5	5	5	15	4	5	3	5	4	5	2	4	32
5	5	5	15	5	1	3	3	1	2	1	3	19
4	5	5	14	4	3	2	3	1	2	1	2	18
3	5	5	13	5	4	3	3	1	2	1	2	21
5	1	5	11	2	1	3	3	2	4	1	2	18
5	5	5	15	5	4	3	3	1	5	1	4	26
5	5	5	15	5	4	2	4	2	5	1	4	27
5	5	5	15	5	2	2	2	2	3	1	3	20
5	5	5	15	3	2	3	3	1	3	1	2	18
5	5	5	15	5	4	4	3	1	3	1	3	24
4	5	5	14	5	5	3	5	5	4	4	4	35
5	5	5	15	5	5	3	5	5	5	5	3	36
5	5	5	15	5	4	3	3	1	3	1	3	23
5	5	5	15	5	3	3	4	2	2	2	4	25
5	5	5	15	4	5	2	3	1	5	2	3	25
5	5	5	15	5	4	2	4	2	3	1	4	25
4	5	5	14	5	3	2	4	2	3	1	4	24
5	5	5	15	5	4	3	4	2	3	1	4	26
5	5	5	15	5	2	3	3	1	3	1	2	20
4	5	5	14	5	5	3	4	1	5	1	4	28
5	5	5	15	5	2	3	3	1	5	1	4	24
5	5	5	15	5	4	3	3	3	3	2	3	26
4	5	5	14	5	3	3	5	3	3	2	3	27
5	5	5	15	5	5	4	5	2	5	3	4	33
5	5	5	15	5	4	4	4	1	5	3	4	30
5	5	5	15	5	5	3	5	4	5	5	4	36

Services Items^4^						​	​	​	​	​	​	​	​	Sub-total	Total	Rating^5^
12	13	14	15	16	17	18	19	20	21	22	23	24	25	​	​	​
5	5	5	3	5	2	2	5	5	5	5	3	2	4	56	102	GOOD
5	5	5	5	5	3	5	5	5	3	5	2	2	5	60	102	GOOD
5	3	5	4	5	1	5	5	5	5	2	2	2	4	53	101	GOOD
5	5	3	3	5	2	5	5	5	4	5	2	4	5	58	104	GOOD
5	5	3	4	3	2	5	5	5	4	5	1	1	4	52	97	FAIR
5	2	3	5	4	2	5	5	5	5	5	1	2	5	54	94	FAIR
3	3	5	5	5	1	5	5	5	3	5	2	1	2	50	92	FAIR
4	3	5	4	5	2	5	5	5	5	1	1	2	5	52	98	FAIR
4	1	5	5	5	3	5	5	5	5	4	2	5	5	59	102	GOOD
2	3	5	5	5	2	4	5	5	4	5	3	3	4	55	99	FAIR
4	4	4	4	5	2	4	5	5	5	5	3	1	4	55	102	GOOD
4	5	5	4	5	4	4	5	5	5	4	3	3	5	61	107	GOOD
4	4	4	4	3	3	4	5	5	5	4	3	1	5	54	101	GOOD
1	2	1	1	1	1	1	1	1	1	1	1	1	4	18	52	NOT IPS
2	3	3	4	3	2	4	1	1	3	3	1	1	4	35	67	NOT IPS
2	4	5	4	3	3	5	1	1	5	3	1	1	5	43	77	FAIR
5	3	3	3	3	1	4	1	1	5	3	2	1	1	36	65	NOT IPS
4	3	3	4	3	1	5	5	5	5	3	2	1	4	48	89	FAIR
4	3	3	4	5	1	5	5	5	2	3	3	1	5	49	91	FAIR
4	2	3	3	3	1	1	1	1	5	1	1	1	3	30	65	NOT IPS
5	4	4	4	3	1	4	1	1	5	3	1	3	3	42	75	FAIR
5	4	4	3	3	1	4	1	1	5	3	1	1	5	41	80	FAIR
5	5	5	5	5	5	5	5	5	4	5	3	1	4	62	111	GOOD
4	5	5	5	5	1	4	5	5	5	5	5	1	4	59	110	GOOD
5	3	3	4	3	2	5	5	5	5	3	1	2	4	50	88	FAIR
4	3	3	3	3	1	4	1	1	5	4	1	1	5	39	79	FAIR
1	2	3	3	1	1	3	1	1	5	3	1	1	4	30	70	NOT IPS
4	2	4	4	3	2	4	1	1	5	1	1	2	5	39	79	FAIR
4	3	4	4	3	2	5	1	1	1	3	1	1	5	38	76	FAIR
3	3	3	4	5	2	4	5	5	5	3	2	3	5	52	93	FAIR
1	2	3	4	3	2	4	5	5	5	1	1	1	4	41	76	FAIR
5	5	4	4	5	4	5	5	5	5	5	5	3	5	65	107	GOOD
4	4	3	2	3	1	5	1	1	5	1	1	1	4	36	75	FAIR
5	5	3	5	5	2	5	5	5	4	5	1	1	3	54	95	FAIR
5	5	5	4	5	2	1	5	5	4	4	3	1	5	54	95	FAIR
5	3	4	5	3	2	5	5	5	5	5	4	1	5	57	105	GOOD
3	3	4	4	3	2	5	4	5	5	5	4	3	5	55	100	GOOD
5	4	5	5	5	4	5	5	5	5	5	4	1	5	63	114	GOOD

1. Each row represents one fidelity review with sites anonymised. 2. Staffing Items - 1. Caseload size; 2. Employment services staff; 3. Vocational generalists. 3. Organisation Items - 4. Integration of rehabilitation with mental health through team assignment; 5. Integration of rehabilitation with mental health through frequent team member contact; 6. Collaboration between employment specialists and Work and Income staff; 7. Vocational unit; 8. Role of employment supervisor; 9. Zero exclusion criteria; 10. Mental health agency focus on competitive employment; 11. Executive team support for employment support; 4. Services Items - 12. Work incentives planning; 13. Disclosure; 14. Ongoing, work-based vocational assessment; 15. Rapid job search; 16. Individualised job search; 17. Job development - frequent employer contact; 18. Job development - quality of employer contact; 19. Diversity of jobs; 20. Diversity of employers; 21. Competitive jobs; 22. Individualised follow along supports; 23. Time-unlimited follow along supports; 24. Community-based services; 25. Assertive engagement and outreach. 5. Source: Bond GR, Peterson AE, Becker DR, Drake RE. Validation of the revised individual placement and support fidelity scale (IPS-25). Psychiatric services. 2012 Aug; 63(8):758-63.

### Micro factors

Wharakura and colleagues [19] identified that frontline collaboration factors including practitioner values alignment, role clarity, beliefs about collaboration and employability alongside information sharing and coordinated, person-centred practice are important between employment, health and welfare services. Available evidence to understand these factors were limited. However, fidelity scale scores do provide some indication of the strength of practitioner collaboration. Item 5 – integration with clinical teams was generally rated very strongly, an indication of collaborative working, although variable scores on zero exclusion also indicate a possible values dis-alignment, where some clinical team members do not offer employment support to everyone on their caseload. Also inconsistent, and indicating an absence of collaboration in some sites, are low scores on Item 6 – collaboration with local Work and Income staff. An important area for improving service quality and outcomes.

### Aotearoa NZ research and evaluation

Local research remains critical to understanding IPS effectiveness, informing policy and supporting national scale-up [18]. Research, led by the Ministry of Social Development, using data from IPS programs, and New Zealand’s Integrated Data Infrastructure confirmed the impact of IPS programs for people in contact with specialist mental health and addiction services, and who are facing significant labour market disadvantage. Results demonstrated outcomes exceeding international benchmarks, and high relative effectiveness across ethnic groups [15].

Cost-effectiveness analysis examining the impact of IPS programs on public services, particularly health, welfare, Inland Revenue and justice, demonstrates the economic case for investment in IPS programs [30]. Over three years, a higher proportion of participants in IPS programs had gained employment than similar individuals who did not go through the program. In addition, income from all sources was higher for IPS participants and they were more likely to gain qualifications. The evaluation also found significant increases in employment and increased mental health service engagement for Māori. These findings align with international evidence of IPS cost-effectiveness [31]. However, in the NZ study, females whilst earning more, were found to have no increase in overall income, which was likely due to a net reduction in welfare benefit payments, reinforcing the need for equity to be addressed across both access and outcomes.

Earlier studies funded by the Health Research Council of NZ [32] also identified the effectiveness and suitability of IPS in the NZ context, and mechanisms for successful integration across health and employment services. Furthermore, a recent research paper, published by the Institute for Democratic and Economic Analysis (IDEA) recommends the completion of the roll-out of IPS, to ensure multifaceted support for jobseekers facing multiple barriers to employment [13].

## Policy options

A systematic examination of the economic and social policy context, during 2017, in New Zealand concluded that the policy context was currently hindering the availability of evidence-based employment practices for adults with mental health conditions. Policy challenges and conflicts, particularly across health and welfare approaches to purchasing and contracting employment support services and the lack of accountability in health service policy to bring employment support into the core business of health treatment services [2]. Two of the policy solutions identified in the review have been actioned: a trial of a coordinated investment approach across health and welfare, and the expansion of the technical assistance program, with the creation of the national centre, Work Counts. However, many of the identified policy challenges remain.

Aotearoa New Zealand stands at a pivotal moment: IPS workforce capacity has doubled, local evidence has proven the impact, but uneven coverage risks deepening inequities if growth stabilises without national coordination and roll-out. Furthermore, funding for implementation support has not grown alongside frontline program expansion and poses risks to sustainability and quality. National scale-up represents a comparatively low-risk investment because effectiveness, implementation infrastructure and workforce capacity already exist.

Equity must be built into systems, not assumed. Accountability for Māori and Pacific participation and outcomes should be embedded in contracts and data frameworks. Recent program expansion into Māori-led services shows what is possible when cultural leadership and local design are enabled.

The IPS authorising environment consists of different channels of policy, funding, and commissioning in parallel, occasionally converging but not yet unified, and certainly not coordinated. This analysis shows there has been limited progress in the IPS authorising environment since the 2017 policy review [2].

Changes are needed across the macro, meso and micro levels to enable and sustain national access and quality of IPS programs (Table 3). Whilst the policy environment is supportive, this is not sufficient for national scale-up. Three core changes are needed [1] Employment needs to be set as a core outcome of specialist mental health and addiction services [2]; There needs to be shared accountability with cross-government investment in the scale-up of evidence-based IPS programs; and [3] Investment in the infrastructure required for high-fidelity IPS implementation at scale (Table 3).

**TABLE 3:** Policy shifts and supporting actions.

Policy shifts	Make employment a core outcome of specialist mental health and addiction services	Establish shared accountability between Health NZ and MSD for employment outcomes of people accessing specialist mental health and addiction services	Invest in the infrastructure required for high-fidelity IPS implementation at scale
Supporting actions			
Macro (national policy)	Include competitive employment as a national mental health outcome measure; incorporate employment indicators into service specifications and accountability frameworks; routinely report employment outcomes alongside clinical outcomes	Develop joint Health NZ and MSD commissioning arrangements; establish shared outcome measures and reporting requirements; create national governance mechanisms for IPS scale-up and implementation. Shared accountability should include accountability for Māori and Pacific access and outcomes	Introduce a national IPS implementation strategy; establish dedicated funding mechanisms; invest in workforce development; implement national fidelity monitoring and benchmarking; strengthen national data collection systems
Meso (organisation/service)	Embed employment goals within treatment planning; integrate IPS into multidisciplinary team practice; implement zero-exclusion referral approaches; routinely review employment outcomes at service level	Formalise partnerships between IPS providers and Work and Income; develop integrated referral and support pathways; establish regional cross-sector planning and oversight mechanisms	Undertake regular fidelity reviews; use quality improvement processes; develop IPS leadership capability; participate in communities of practice; benchmark service performance
Micro (in practice)	Discuss employment aspirations early; include employment goals in recovery planning; provide work-focused support regardless of diagnosis, symptoms, or perceived readiness	Promote coordinated support between IPS specialists, clinical teams and Work and Income staff; provide integrated employment and benefits planning; ensure seamless transitions between welfare support and employment	Provide structured supervision and training; strengthen employer engagement capability; support fidelity-informed practice improvement; invest in continuing professional development

At a meso level, it is crucial that health leaders and managers in community specialist services build the organisational commitment to integration of employment support as a core element of health service delivery, embedding employment goals within treatment planning. At the micro, practitioner level, more can be done to discuss employment aspirations early in treatment, engage with employers, provide ongoing in‐work support, and build stronger working relationships with welfare services.

The next phase for IPS growth demands coherence, stability, cross-government and cross-sector commitment to make an equitable and sustainable shift.

## Conclusion

This policy analysis demonstrates that IPS has become an established component of employment support in Aotearoa New Zealand, with multiple sites achieving good fidelity and employment outcomes among populations facing substantial barriers to employment. However, considerable variation in IPS coverage and in fidelity indicates that access to high-quality IPS remains uneven and dependent on local conditions rather than a consistently supported national approach.

The fidelity findings suggest that the main barriers to IPS scale-up are no longer primarily clinical or workforce-related. Rather, lower-scoring items were concentrated in areas requiring system-level coordination, including organisational leadership, cross-agency collaboration, employer engagement, and community-based support. These findings indicate that implementation challenges increasingly reflect features of the policy and commissioning environment rather than limitations of the IPS approach.

Achieving equitable access to high-fidelity IPS will require three interdependent policy shifts. First, employment should be recognised and measured as a core outcome of specialist mental health and addiction services. Second, accountability for employment outcomes should be shared across Health NZ and the Ministry of Social Development through joint commissioning and governance arrangements including accountability for equitable access and outcomes. Third, sustained investment is required in the implementation infrastructure that supports IPS delivery, including workforce development, fidelity monitoring, quality improvement systems, data capability, and technical assistance.

Access to employment, participation, and social inclusion is recognised as a human right, and evidence-based employment support can help realise those rights [33]. Returning to, or commencing, employment is also an important aspiration for many people accessing mental health and addiction services [6]. New Zealand already possesses the evidence, service expertise, workforce capability and implementation experience required for IPS scale-up. The central challenge is no longer whether IPS works, but whether public agencies are prepared to establish the accountability, shared commissioning and implementation supports required to make access to employment a routine component of mental health care.

IPS remains the internationally recognised evidence-based approach for supporting people with mental health conditions into competitive employment. It is simultaneously an employment and a mental health intervention, as well as a mechanism to address systemic inequities. New Zealand does not need more evidence that IPS works. It needs a system that is accountable for delivering at scale.
